# Improving image accuracy of region‐of‐interest in cone‐beam CT using prior image

**DOI:** 10.1120/jacmp.v15i2.4628

**Published:** 2014-03-06

**Authors:** Jiseoc Lee, Jin Sung Kim, Seungryong Cho

**Affiliations:** ^1^ Department of Nuclear and Quantum Engineering Korea Advanced Institute of Science and Technology Daejeon Korea; ^2^ Department of Radiation Oncology Samsung Medical Center, Sungkyunkwan University School of Medicine Seoul Korea

**Keywords:** image accuracy, prior image, region of interest, cone‐beam CT

## Abstract

In diagnostic follow‐ups of diseases, such as calcium scoring in kidney or fat content assessment in liver using repeated CT scans, quantitatively accurate and consistent CT values are desirable at a low cost of radiation dose to the patient. Region‐of‐interest (ROI) imaging technique is considered a reasonable dose reduction method in CT scans for its shielding geometry outside the ROI. However, image artifacts in the reconstructed images caused by missing data outside the ROI may degrade overall image quality and, more importantly, can decrease image accuracy of the ROI substantially. In this study, we propose a method to increase image accuracy of the ROI and to reduce imaging radiation dose via utilizing the outside ROI data from prior scans in the repeated CT applications. We performed both numerical and experimental studies to validate our proposed method. In a numerical study, we used an XCAT phantom with its liver and stomach changing their sizes from one scan to another. Image accuracy of the liver has been improved as the error decreased from 44.4 HU to −0.1 HU by the proposed method, compared to an existing method of data extrapolation to compensate for the missing data outside the ROI. Repeated cone‐beam CT (CBCT) images of a patient who went through daily CBCT scans for radiation therapy were also used to demonstrate the performance of the proposed method experimentally. The results showed improved image accuracy inside the ROI. The magnitude of error decreased from −73.2 HU to 18 HU, and effectively reduced image artifacts throughout the entire image.

PACS number: 87.57. Q‐

## I. INTRODUCTION

Patient dose in diagnostic follow‐ups of disease, such as quantitative assessment of fat content in liver or calcium scoring in kidney, is relatively high when compared to standard CT scans because of their repeated scans. Dose reduction has been a major concern of radiation medical applications. Reducing patient dose by decreasing the tube current has been suggested by several investigators.^1^, ^2^, ^3^ This approach may increase image noise, which often deteriorates the overall image quality and decreases the image accuracy of the ROI considerably. A more sophisticated method adapts the tube current to X‐ray attenuation as it changes with projection angle.^4^, ^5^, ^6^ The idea is to lower the tube current for projections associated with relatively low attenuation. On top of these techniques, an additional dose reduction scheme would be desirable, particularly considering the repeated CT scans. Diagnostically significant information is often concentrated in a relatively small region of interest (ROI) in a reconstructed 3D image. Thus, region‐of‐interest imaging technique is considered a reasonable dose reduction method in which the ROI is only illuminated during a scan by use of a filter that blocks the beam outside the ROI. Through this method, all the X‐ray photons penetrating the region of interest are not affected by the ROI filter. The projection data acquired by the ROI imaging technique are truncated, similar to a case when a patient's body extends outside the scanning field of view (FOV). Truncation artifacts and loss of image accuracy inside the ROI would occur when truncated projection data are directly used for image reconstruction by use of the filtered backprojection (FBP) algorithm, which is the most widely used algorithm in the commercial CT scanners. Thus, the accuracy of the reconstructed image of the ROI by the FBP algorithm may be insufficient especially for quantitative assessment.^7^, ^8^, ^9^, ^10^, ^11^, ^12^, ^13^

In the past, there have been several studies on artifact reduction in 3D images reconstructed from truncated data.^14^, ^15^, ^16^, ^17^, ^18^, ^19^, ^20^, ^21^, ^22^ Most of the reported methods estimated the incomplete part without any prior information. As a result, the image artifacts are reduced outside the ROI to a certain degree, depending on the severity of data truncation. In 2002, Ruchala et al.^(23)^ reported an algorithm that utilizes a planning CT data as a priori information, and reduces image artifacts of online CT caused by the limited field of view. Their study, however, focused on improving the image quality of the overall anatomy particularly in the outside ROI, and not much attention has been paid to the accuracy of the inner ROI. Although various interior tomography techniques have also been investigated under certain assumptions, image accuracy of the ROI in general is not guaranteed due to the mathematical instability of the interior problem.^24^, ^25^, ^26^, ^27^ The aim of our study is to reconstruct an accurate ROI image for quantitative assessment by use of a priori CT scan data. We used projections of cone‐beam CT that have been initially acquired from a patient without using the ROI filter, and synthesized new data combining the ROI projections currently acquired with the previous data. Therefore, substantial dose reduction is expected by use of the proposed method, where a conventional CT covering the entire cross section is just required once initially out of the repeated scans. Usually, patient anatomy outside the ROI is relatively less changing in its shape and volume. For example, the bony structures are hardly changing in time of the repeated scans compared to other organs, such as kidney or liver, particularly considering the change of organs after appropriate treatments. Therefore, we used prior data of the same patient for estimating the outside ROI of postacquisitions. As a feasibility test, we implemented a computer simulation with nonuniform rational B‐spline (NURBS)‐based eXtended CArdiac‐Torso (XCAT) phantom and the XCAT‐based CT projector.^28^, ^29^ We prepared two phantoms of different sizes of liver and stomach to represent changes within the ROI between acquisitions. Cone‐beam CT data were accordingly acquired by use of the two phantoms, assuming that one dataset is prior data and the other dataset current data. To synthesize the projection data, it is required to register the two reconstructed images: one from a prior scan and the other ROI image from a current truncated scan. For image registration between the images reconstructed from the two datasets, we used a normalized mutual information method.^30^, ^31^, ^32^ In the simulation study and in the experiment, we assumed that the ROI filter perfectly blocks the incident X‐rays. In other words, pixel values outside of the ROI were set to zero. Unlike the previously reported methods, we used the prior data from the same patient to estimate the blocked region. Thus, the data information outside the ROI is expected to have more reliable anatomic information than other methods.

## II. MATERIALS AND METHODS

### A. Simulation study

**Figure 1:**
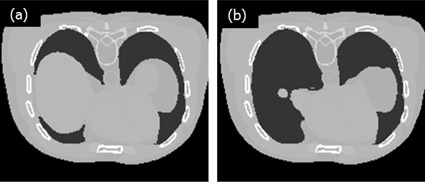
Slices of the (a) prior and (b) current phantoms with half of the original liver and stomach volumes reduced.

**Figure 2:**
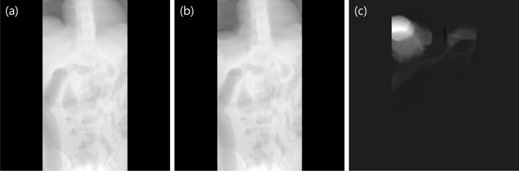
Projections of (a) a priori data with zero padding outside the ROI, (b) ROI data, and (c) the difference between (a) and (b).

**Figure 3:**
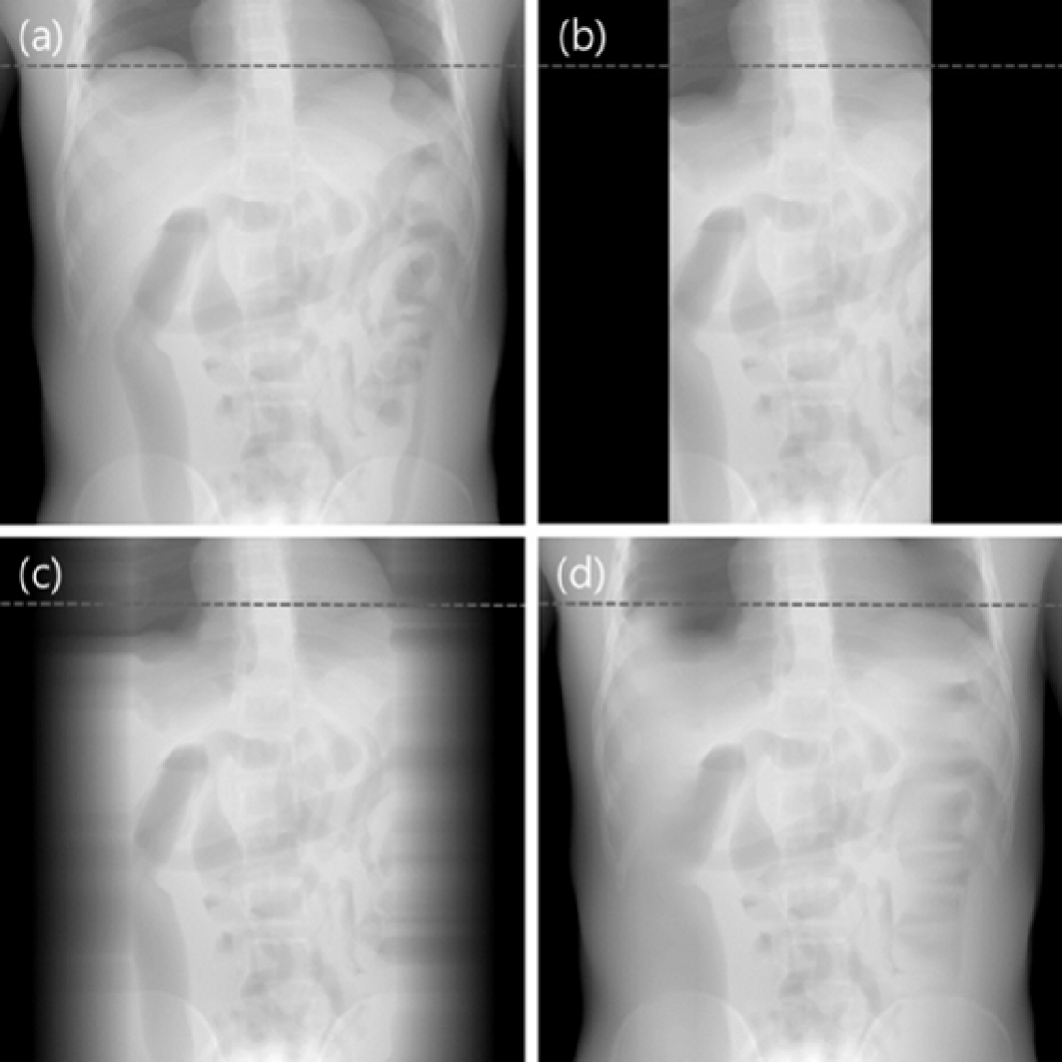
Projections of reference and three schemes: (a) prior, (b) ROI, (c) linear extrapolation, and (d) the proposed method.

We performed simulations to test a feasibility of our proposed method in a chest CT. Two numerical phantoms were prepared with the XCAT computer simulation tools. One of the phantoms served as a priori data, while the other represents a current anatomy including volume changes of organs; the liver and stomach sizes have been reduced to half of the original. One can see the difference in the organ volumes of the two simulated phantoms in a slice as shown in Fig. 1. Numerical values of the liver and the stomach volumes are summarized in Table 1. CT projections of the two phantoms were acquired using the following geometry parameters: source‐to‐axis distance 1510 mm, source‐to‐detector distance 1800 mm, detector size 410×410 mm, the number of projections 360. It was assumed that the width of the ROI filter opening is a half of the total field of view (FOV). The filters were then set axially at each side of the FOV. Furthermore, we assumed that the filters perfectly block all incident X‐rays. Hence, pixel values in the shielded areas are set to zero, as shown in Fig. 2. Three different schemes were tested for image reconstruction: using the ROI data only, using the ROI data with a linear extrapolation, and using the ROI data with a priori data. The example projection images of the different schemes are shown in Fig. 3. The first scheme, that uses data from the ROI only, is expected to generate truncation image artifacts in the 3D reconstructed image. Nevertheless, it was used as a reference with which to compare the accuracy and consistency of the other schemes. The second scheme uses a simple linear extrapolation, and no variation of anatomical information is reflected outside the ROI. Even though this scheme is simple, it is reported to reduce artifacts by inserting such pseudodata. The third scheme, ROI with a priori data, is the proposed method in this paper. It involves the use of the ROI data plus a priori data of the same patient which has anatomical information outside the ROI. The ROI was not directly fitted to the prior data. A gap was made at the boundaries, and interpolation between the ROI and the outside ROI of the prior data was done in the projections. A smoothing filter was also applied to make a smooth transition. For image reconstruction, FBP algorithm was used.

**Table 1:** Volume of liver and stomach in a prior and ROI image (ml).

	*Prior*	*ROI*
Liver	1631.3	809.75
Stomach	399.33	202.60

### B. Experimental study

In order to compare the performance of the proposed method with other techniques, the schemes previously mentioned were applied to real human pelvic cone‐beam data obtained at different times in an image‐guided radiation therapy, IGRT (Novalis Tx, Varian Medical System, Palo Alto, CA and BrainLAB, Feldkirchen, Germany). For the first day data, we used projections directly for image reconstruction. The following day data were numerically truncated to simulate ROI imaging. Image registration between the first day CT image and the following day CT image of the ROI was successfully conducted by use of the normalized mutual information method. Numerical projection data were synthesized from the first day CT image after registration, and they were combined with the following day data to generate truncation‐corrected data. Three different schemes that were introduced in the previous section were applied to the human cone‐beam CT projections. Again, the FBP algorithm was used for 3D image reconstruction.

## III. RESULTS

### A. Simulation study

**Figure 4:**
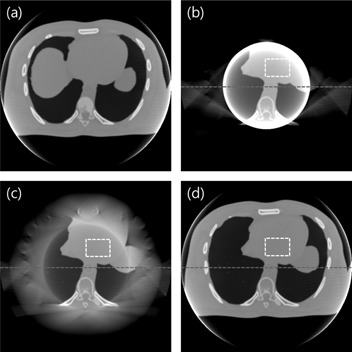
Reconstructed images of a priori schemes: (a) prior, (b) ROI, (c) linear extrapolation, and (d) the proposed method. Display window: [‐200 400] HU.

**Figure 5:**
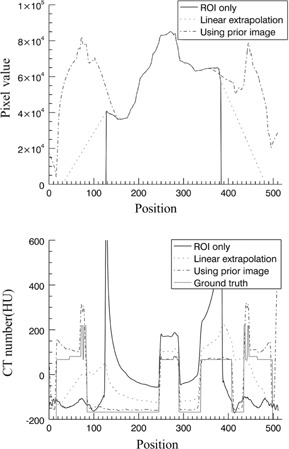
Line profiles of the projections are shown in (a) and those of the reconstructed images in (b).

This study focused on the thoracic‐abdominal region, specifically, the area surrounding the liver and the stomach. The position and volume of the organs between acquisitions, except for the stomach and the liver, remain unchanged. Projections from a priori and ROI acquisitions are shown in Fig. 2. In Fig. 2(a), outside the ROI of a prior data was made to have zero value for comparing the organ volumes in the prior ROI with the ROI data. The difference of the pixel values around the liver and the stomach is presented in Fig. 2(c). Projections of the three estimation schemes are presented in Fig. 3. Having no truncations, the reference data shown in Fig. 3(a) was used as a priori data. The ROI data in Fig. 3(b) contains completely blocked projections outside the ROI and without any estimation schemes applied. The projections outside the ROI in Fig. 3(c) were linearly extrapolated and have no anatomical information related to the ROI. Lastly, the proposed method was implemented, and the synthesized projection is shown in Fig. 3(d). Discontinuity of data in the synthesized projections was reduced by using IDL (Exelis Visual Information Solutions, Boulder, CO) built‐in functions (e.g., smooth and interpolation). The data prepared from the three estimation schemes were reconstructed using the FBP algorithm. In Fig. 4(a), the reconstructed image from the reference data is shown. In Fig. 4(b), severe artifacts near the ROI edges were observed in the reconstructed image from the truncated ROI data. Moreover, no useful anatomical information is found outside the ROI. In Fig. 4(c), image artifacts around the ROI have been alleviated due to the linearly extrapolated projections outside the ROI. No anatomical information is found outside the ROI in this case, as well. In Fig. 4(d), it is observed that the image artifacts near the ROI edges in the lung region have been nearly completely removed by use of the proposed method. The line profiles along a dashed line in Fig. 3 are shown in Fig. 5(a). The ROI image accuracy can be compared from the line profiles of the reconstructed images, as shown in Fig. 5(b).

Only the proposed method shows meaningful information outside the ROI. More importantly, the image accuracy of the ROI is greatly preserved in the image reconstructed by use of the proposed method. The thin line represents the ground truth which is nothing but the numerical phantom itself as a reference. The line profile of the reconstructed image of the ROI data only is plotted in a thick line. Not only does it show the image artifacts around the ROI, but also inaccuracy of the pixel values inside the ROI is evident. Notice that the pixel values inside the ROI have been significantly increased because the truncated data cause data enhancement when it goes through a filtering process of the FBP algorithm. The line profile of the reconstructed image with the linear extrapolation of data outside the ROI is plotted in a dotted line. The image artifacts near the ROI edges are substantially suppressed and the image pixel values have been decreased compared to the ROI‐only case. However, a significant level of data enhancement inside the ROI still remains. The line profile of the reconstructed image by use of the proposed method is plotted in a dash‐dot line. Image accuracy of the ROI has been greatly improved compared to others.

As an attempt of quantitative assessment of image accuracy, the average CT numbers inside the ROI, marked by the white dotted square in Fig. 4, are summarized in Table 2. The average CT number of the ROI‐only image is about 149 HU higher than that of the ground truth. This can be explained by the pixel value enhancement due to incomplete data in the ROI imaging. The average CT numbers of the image reconstructed from the linearly extrapolation method is higher than the ground truth. The error is quite reduced to about 44.4 HU, though. The average attenuation value of the image reconstructed by use of the proposed method is very close to the ground truth; the error is practically negligible. This result strongly supports a feasibility of the propose method for quantitative imaging of an ROI.

**Table 2:** Average CT numbers inside the ROI of reconstructed images for the simulation study

	*ROI*	*Linear Extrapolation*	*Proposed Method*	*Ground Truth*
Ave. CT #	233	128	83.5	83.6
Error (±)	149	44.4	−0.1	N/A

### B. Experimental study

**Figure 6:**
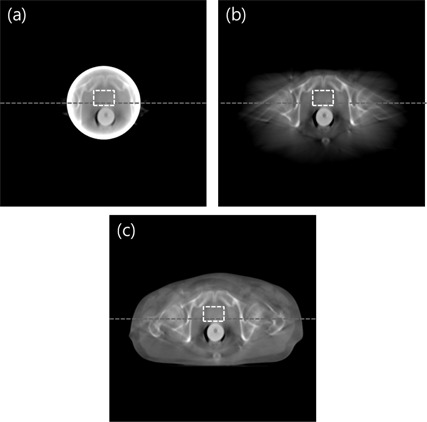
Reconstructed images of three schemes: (a) ROI, (b) linear extrapolation, and (c) the proposed method. Display window: [‐500 1500] HU.

**Figure 7:**
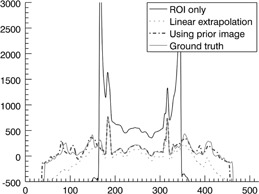
Line profiles of the reconstructed images along the dashed lines shown in Fig. 6.

Three estimation schemes were also applied to the real patient pelvic cone‐beam CT data acquired from Samsung Medical Center in Seoul, South Korea. In contrast to the simulation test, a dense object is included inside the ROI, as shown in Fig. 6. The object at the center of the image represents a rectal balloon which is inserted for minimizing organ motion. The reconstructed image from the ROI‐only data is presented in Fig. 6(a). As expected, a strong ring artifact appears in the image and a data enhancement is also observed in Fig. 7. The reconstructed image from the linearly extrapolated data outside the ROI is shown in Fig. 6(b). The ring artifact and data enhancement inside the ROI appear to be slightly reduced, whereas meaningful anatomical information is still missing outside the ROI. In this case, the pixel values of the ROI are slightly lower than the ground truth, which is the original CT image in the following scans. This is due to over‐estimation of the outside ROI data by use of a linear extrapolation in this example. The reconstructed image by use of the proposed method is shown in Fig. 6(c). Compared to the other schemes, the image artifacts are greatly suppressed, and the reconstructed image closely resembles a general diagnostic image. The line profiles of the reconstructed images along the dotted line in Fig. 6 are plotted in Fig. 7.

Inside the ROI, the linearly extrapolated data is close to the ground truth, while data from the proposed method present almost the same pixel values as the ground truth. Outside the ROI, pixel values from the proposed method is similar to the ground truth. This means that the anatomical information outside the ROI may remain almost unchanged throughout the radiation therapy procedure.

As a quantitative assessment of image accuracy, the average CT numbers inside the ROI, marked by the white dotted square, are summarized in Table 3. The average CT number of the ROI‐only image is about 362 HU higher than the average values of the ground truth. The average CT value of linear extrapolation method is lower than the ground truth. The error is about −73.2 HU. The average CT value of the proposed method is very close to the ground truth. Particularly, the error is about 18 HU. This implies that the proposed method can reconstruct quantitatively accurate ROI image at reduced dose in repeated CT applications.

**Table 3:** Average pixel values inside the ROI of reconstructed images for the application study

	*ROI*	*Linear Extrapolation*	*Proposed Method*	*Ground Truth*
Ave. CT #	489	53.8	145	127
Error (±)	362	−73.2	18	N/A

## IV. DISCUSSION

In this paper, we proposed a method using a priori data similar to the one presented in Ruchala et al.^(23)^ The focus of that work was on extending the virtual FOV to cover the entire body in the transverse direction for the purpose of calculating the dose distribution based on the extended image. Also, image accuracy of the ROI is not guaranteed generally in various interior tomography techniques, which have been studied under certain assumptions due to the mathematical instability of the interior problem.^24^, ^25^, ^26^, ^27^ In contrast, our focus is on improving the image accuracy of the ROI for certain clinical applications which may require accurate image information, such as density analysis of the liver. Thus, the anatomic data outside the ROI in a reconstructed image is considered as reasonably reliable, although anatomical change to some degree is inevitable. Discontinuities in projection images are potentially the causes of artifacts in the reconstructed 3D images. To mitigate the artifacts, we defined a gap, interpolated between the outside ROI of the prior image and ROI image, and formed smoothly connected edges of the ROI. The proposed method showed a good performance in suppressing both the ring artifact and the data enhancement inside the ROI.

The success of the proposed approach depends on the availability of a priori CT data of a patient. Therefore, clinical applications may find it useful, such as perfusion study, prognostic evaluation, and daily CBCT for IGRT, particularly when quantitative image information is important. In these applications, ROI imaging may lead to a substantial decrease of the patient radiation dose in successive image acquisitions by shielding outside the ROI. The degree of dose reduction largely depends on how big the size of ROI is. In our experiment, we assumed that the ROI size is about half of the FOV size and that the filter absolutely stops the X‐ray beam. Under this condition, the total amount of exposure through the ROI aperture would be roughly half of the full exposure. Similarly, overall imaging radiation dose to the patient would be accordingly decreased, although a local distribution of dose may be more complicated. Assessment of dose distribution will be included in our future study. Monte Carlo simulation tools can be utilized for such an assessment study, and the study can be further extended to phantom‐based dosimetric experiments.

## V. CONCLUSIONS

ROI imaging technique in repeated CT scans has merits in decreasing the integral dose by shielding X‐ray radiation outside the ROI. However, image artifacts and decrease in the image accuracy may result from incomplete data, thereby hampering its application as a method of quantitative imaging. We have successfully demonstrated the feasibility of an ROI imaging method using a priori patient data for low‐dose quantitative imaging through both numerical and experimental studies.

## ACKNOWLEDGMENTS

This work was supported in part by the NRF grant NRF‐2013M2A2A9043476, and by the MEST grant R0001270 and R0001376 in Korea. The authors are very thankful to Kihong Son and Rizza D. Pua for their discussion and help.
